# Bleeding Meckel's Diverticulum in a 33-Year-Old Female Diagnosed with Video Capsule Endoscopy and a Technetium-99 m Pertechnetate Scan with a Favorable Response to H_2_ Blocker and PPI

**DOI:** 10.1155/2021/1381395

**Published:** 2021-12-09

**Authors:** Gregor Krstevski, Urim Isahi, Vladimir Andreevski

**Affiliations:** University Clinic of Gastroenterohepatology, Medical Faculty, Ss. Cyril and Methodius University, Boulevard Mother Teresa 17, Skopje 1000, North Macedonia

## Abstract

Meckel's diverticulum is a true diverticulum consisting of all three layers of the small intestine resulting from incomplete regression of the vitelline duct. While it is often benign, it can present with serious complications such as intussusception, ulceration, torsion, hemorrhage, obstruction, inflammation, and fistula formation. Although it typically presents in infancy and early childhood, it can also manifest much later into adulthood. We report a case of Meckel's diverticulum complicated by significant bleeding in a 33-year-old female patient. Diagnosis was accomplished with video capsule endoscopy and a technetium-99 m pertechnetate scan. The patient responded well to acid suppression, initially with an H_2_ blocker and later with a PPI (proton pump inhibitor), and remained asymptomatic for nearly four months in the interim to definitive surgical treatment. Microscopic examination of the resected diverticulum confirmed the presence of ectopic gastric mucosa. A PubMed literature search revealed several similar cases of Meckel's diverticulum complicated by hemorrhage with a favorable response to H_2_ blockers and PPIs. While surgical resection remains the mainstay of definitive treatment, medications aimed at acid suppression can delay the need for urgent surgery, allow for diagnostic assessment, and optimize conditions for elective surgical treatment.

## 1. Introduction

Meckel's diverticulum is the most common congenital anomaly of the gastrointestinal tract. It is a true diverticulum that results from incomplete regression of the omphalomesenteric, also known as the vitelline duct. Although it is often benign, it can cause complications, and these include intussusception, ulceration, torsion, hemorrhage, obstruction, inflammation, and fistula formation. The rule of 2s has traditionally been used to characterize this condition, referring to a population prevalence of 2%, typically located within 2 feet proximal to the ileocecal valve; symptomatic lesions largely present prior to the age of 2, and their length is often approximately 2 inches (5 cm). Nonetheless, Meckel's diverticulum can remain silent and become symptomatic in adulthood [1]. We report a case of Meckel's diverticulum complicated by significant bleeding in an adult female diagnosed with video capsule endoscopy and confirmed with a technetium-99 m pertechnetate scan that responded well to acid suppression in the interim prior to surgical resection.

## 2. Case Report

**Figure 1:**
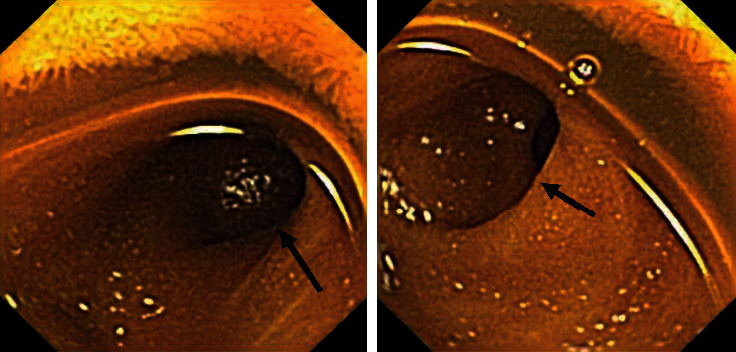
Video capsule endoscopy demonstrated a solitary diverticulum (indicated by black arrows) in the distal half of the ileum.

Our patient is a 33-year-old female that presented with a three-day history of abdominal pain, malaise, and maroon-colored loose stools. She noted bloating and nonspecific abdominal pain localized in the periumbilical region and epigastrium. During the previous day, she had experienced a syncopal episode at her place of employment with spontaneous return of consciousness. Subsequent laboratory testing revealed anemia with erythrocyte count 2.6 × 10^12^/L, hemoglobin 82 g/L, and hematocrit 23.3%, and urgent upper gastrointestinal endoscopy did not display a bleeding lesion. The patient reported no pertinent past medical history and did not take NSAIDs or blood thinners. She reported no allergies to food or drugs and did not smoke cigarettes, drink alcohol, or use illicit drugs. Family history was positive in her father for hemorrhoids and in her aunt for a duodenal ulcer. On physical examination, blood pressure was 120/70 mmHg, pulse was 70/min, and palpation elicited light pain in the left hemiabdomen. Digital rectal examination showed maroon-colored stool. Additional laboratory testing showed leukocytosis 20.1 × 10^9^/L, prothrombin time 14.3 seconds, INR 1.33, serum amylase 148 U/L, and a normal level of urea, thrombocytes, proteins, and electrolytes. The patient was admitted and transfused with two units of packed red blood cells. She was also treated with an NPO regimen, intravenous fluids, continuous octreotide infusion, and H_2_ blocker. Following treatment, the patient's condition stabilized and no new bloody stools were noted. Laboratory values also trended towards normal. Abdominal ultrasound and repeat upper gastrointestinal endoscopy showed no abnormalities. Lower gastrointestinal endoscopy permitted evaluation up to 10 cm proximal to the ileocecal valve and displayed no significant abnormalities. Video capsule endoscopy (using PillCam SB capsule, Given Imaging, Yoqneam, Israel) demonstrated the presence of a solitary diverticulum in the distal half of the ileum, a finding suggestive of Meckel's diverticulum. Images can be found in Figure 1.

**Figure 2:**
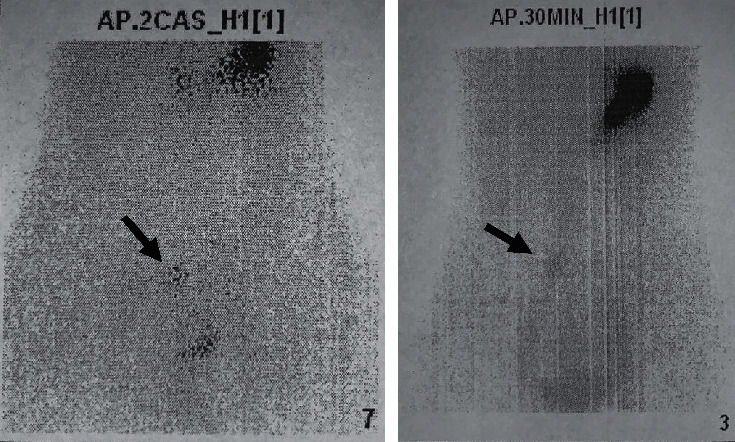
Abdominal scintigraphy with 99 m Tc pertechnetate (Meckel scan) showed abnormal accumulation of the radioisotope indicating the presence of ectopic gastric mucosa. The black arrows point to the areas of abnormal accumulation.

MR enterography did not show any abnormalities; however, abdominal scintigraphy with Technetium-99 m pertechnetate revealed abnormal accumulation of the radioisotope in the distal ileum in correlation with the presence of ectopic gastric mucosa (Figure 2).

**Figure 3:**
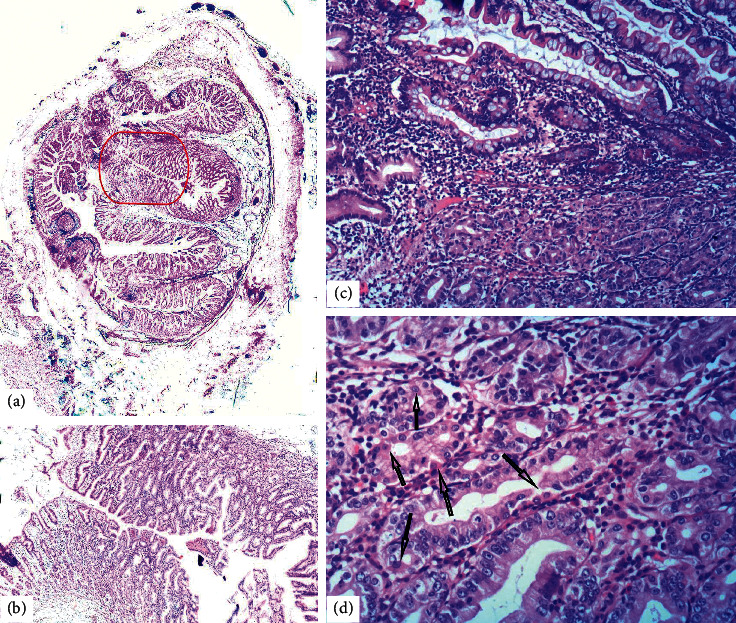
(a) HeEo (x5): cross section of the diverticulum. Gastric mucosa is delineated with a red line. (b) HeEo (x40): the section with gastric mucosa at higher magnification. (c) HeEo (x100): margin between intestinal (upper half) and gastric mucosa (lower half). (d) HeEo (x200): some of the parietal cells in the gastric mucosa (arrows), with a deeply eosinophilic cytoplasm as opposed to the mucinous and chief cells.

The patient was maintained on PPI for nearly four months and remained asymptomatic, with stable hemoglobin and no new episodes of gastrointestinal bleeding. She then underwent laparoscopic surgery, and the diverticulum was resected. The surgical specimen, which showed a diverticulum contralateral to the mesentery, was then sent for histopathologic analysis. The relevant macroscopic and microscopic findings detailed in the pathology report are summarized in Table 1. At high magnification (x200) of the area of gastric mucosa, parietal cells with a deeply eosinophilic cytoplasm in comparison to the mucinous and chief cells are seen (Figure 3).

## 3. Discussion

In summary, this is a case of Meckel's diverticulum in a 33-year-old female patient with significant bleeding and a resultant profound, life-threatening anemia. As noted above, Meckel's diverticulum is a vestigial remnant of the omphalomesenteric duct. It develops in the fourth week of gestation and serves as a connection between the loops of the primitive gut and the yolk sac. It normally obliterates by the tenth week of gestation; however, it can persist even into adulthood. There are several distinct entities that arise from persistence of the vitelline duct. An umbilicoileal fistula occurs when the duct remains entirely patent. In the case of persistence of the distal portion at the umbilicus, it is termed an umbilical sinus. Persistence of the middle portion results in the formation of fluid-filled cysts termed enterocystomas. The most common anomaly is nonobliteration of the proximal segment in continuity with the small intestine, forming what is known as Meckel's diverticulum. It is a true diverticulum consisting of all three layers of the small intestine [2].

The first description of this condition by Johann Friedrich Meckel dates more than 200 years back. In-depth understanding and definition of the embryological origins, histological constitution, and span of complications have been defined since. As pluripotent cells line the vitelline duct, ectopic tissue is a typical feature of Meckel's diverticulum. Gastric mucosa is most common; however, duodenal, colonic, pancreatic, Brunner's glands, and hepatobiliary and endometrial mucosa can also be found. Of importance is that silent or asymptomatic Meckel's diverticula are less likely to contain ectopic tissue [3–5].

Most studies place the true incidence of this abnormality between 0.6% and 4% and the male to female ratio of symptomatic disease at 1.5–4:1. More than half of symptomatic cases requiring surgery occur in children, and the most common complications are obstruction, hemorrhage, and inflammation. This holds true in both the pediatric and adult populations, with obstruction accounting for a higher proportion in children when compared to adults. Bleeding can result from erosion of the adjacent intestinal mucosa by gastric mucosal secretions in the manner of peptic ulceration. Bleeding can also occur in the setting of strangulation, torsion, or intussusception, and these are commonly accompanied by pain. Obstruction can be caused by intussusception, volvulus, herniation, diverticulitis, bowel loop entrapment, and phytobezoar formation. Meckel's diverticulum has a greater neoplastic potential than the remainder of the ileum, and the mean age for Meckelean cancers is 60. Inflammation is another important complication, and it can lead to perforation. The mechanism generally includes blockage of the opening of the diverticulum by food, foreign bodies, large microorganisms such as parasites, and enteroliths. Consequent perforation leads to peritonitis, and the presentation is very similar, if not indiscernible, to acute appendicitis [3–5].

Diagnosis is particularly challenging in adults as Meckel's diverticulum is very rare and mimics an array of differing pathologies [4]. Conventional techniques such as x-ray, ultrasound, CT, and barium studies have limited diagnostic value as the findings are nonspecific. For example, a plain abdominal x-ray may show pneumoperitoneum in the case of perforation or bowel distention proximal to the site of obstruction. Ultrasound may show bowel wall thickening, focal findings that suggest an abscess, or the presence of anomalous vessels via use of Doppler. These findings may raise suspicion, but do not pinpoint to the exact diagnosis. Imaging with labelled red blood cells and angiography of the abdominal blood vessels (the superior mesenteric artery in particular) has greater use as it can pinpoint the exact location of hemorrhage [5, 6]. However, studies demonstrate that angiography is only effective when bleeding exceeds 0.5 ml/min [4]. CT and MR enterography can also provide a good means for the examination of irregularities within the intestinal lumen, wall, and surroundings. The special diagnostic value of the technetium-99 m pertechnetate scan is in the radioisotope's proclivity to gastric mucosa, which is why it has been termed the Meckel scan. The rate of false negatives can be reduced by administering pentagastrin or an H_2_ blocker prior to the scan. These agents increase uptake and decrease the release of the radioisotope by gastric mucosa, respectively. A more novel approach to diagnosing Meckel's diverticulum is direct visualization via capsule endoscopy and anterograde and retrograde double balloon enteroscopy. These allow for more precise localization and characterization of the lesion. Nevertheless, Meckel's diverticulum is most commonly discovered incidentally during surgical interventions for unrelated conditions [5, 6].

Management of incidentally discovered Meckel's is controversial. The lifetime incidence of complications of Meckel's diverticula is approximated at 4–6%. While surgical resection may prevent future adverse events, surgery in itself carries a risk for immediate and delayed complications. When incidentally discovered intraoperatively, weighted risk scores can be applied to reach a decision. Factors that increase the risk for future complications provide support for resection: age (<45 or <50 depending on the author), male gender, lesions 2 cm or longer, broad based, the presence of abnormal tissue, and attached fibrous bands [5]. The surgeon should take into account the patient's comorbidities, other intraoperative strains and complications, and an initial poor prognosis. Therefore, resection of an intraoperatively and incidentally discovered Meckel's diverticulum is largely at the operator's discretion [4–6].

The most common ectopic tissue found in Meckel's diverticula is gastric mucosa. Secretions from these cells can damage and erode adjacent intestinal mucosa, closely resembling peptic gastric and duodenal ulceration. Proton pump inhibitors block the H+/K+ ATP proton pump, thereby inhibiting the final process of acid production and secretion that is mediated by gastrin, H_2_, and cholinergic pathways. H_2_ blockers specifically act on H_2_-dependent acid generation and secretion [7]. It is theorized that acid secretion from Meckel's diverticula that contain ectopic gastric mucosa can be suppressed by the use of PPIs and H_2_ blockers, thereby preventing mucosal damage. Using PubMed, we performed a literature search and looked for articles containing the term Meckel's diverticulum in combination with PPIs, pantoprazole, omeprazole, lansoprazole, H_2_ blockers, ranitidine, famotidine, and cimetidine. The resulting lists of articles were then carefully assessed, with the majority focusing on the use of H_2_ blockers in increasing the sensitivity of the technetium-99 m pertechnetate scan. Several case reports discussing the use of PPIs and H_2_ blockers as treatment modalities for Meckel's diverticulum were identified, and an additional article published in the American Journal of Gastroenterology was identified by using Google search. The relevant details of each case are listed in Table 2. A study by Schonsberg et al. discussing two cases of Meckel's diverticulum that presented with enterorrhagia and were managed with H_2_ blockers was not included as the full text of the study was inaccessible and an attempt at contacting the authors to obtain it was unsuccessful.

Temporary symptom resolution was accomplished with acid suppression in seven of the eight identified cases of bleeding Meckel's diverticulum. Albeit in two of the cases (6 and 7), the diagnosis was highly likely, it was not confirmed with an isotope scan or microscopically. Moreover, case 7 showed the presence of a jejunal angioectasia that was not bleeding at the moment of double-balloon enteroscopy and was treated with argon plasma coagulation. In case number 8, the patient had persistent copious bleeding despite intense acid suppression and ectopic gastric mucosa later being demonstrated microscopically. This suggests that acid suppression with PPIs or H_2_ blockers may be useful in many cases of bleeding Meckel's diverticulum with ectopic gastric mucosa in the interim to definitive surgical treatment.

## 4. Conclusions

To conclude, this case demonstrates that Meckel's diverticulum can manifest with life-threatening blood loss even in the adult population. The practicing gastroenterologist should include this condition in the differential diagnosis of any case of obscure gastrointestinal bleeding and take the necessary diagnostic measures to confirm or exclude its presence. Acid suppression may favorably alter the disease course of Meckel's diverticulum when ectopic gastric mucosa is present, allowing for diagnostic work-up and optimizing conditions for definitive elective surgical treatment. Considering the fact that gastric mucosa is the most common type of ectopic tissue found in Meckel's diverticulum, PPI or an H_2_ blocker should be administered when faced with obscure gastrointestinal bleeding.

## Figures and Tables

**Table 1:** Pathology report of the resected tissue.

Macroscopic findings	We received a partially longitudinally resected segment of the small intestine with a total length of 5 cm, perimeter of 3.5 cm, and mural width of 0.4–0.8 cm. The contour of the small intestinal mucosa is regular, while in a zone of approximately 2.2 × 2 cm contralateral to the mesentery, there is a semicircular widening of the lumen, a diverticulum, with thinning of the wall to 0.4–0.5 cm, and flattened and hyperaemic mucosa. The width of the wall in the remaining part is 0.6–0.8 cm

Microscopic findings	In part, the structure of the small intestinal wall has a regular histologic architecture, with scant chronic inflammatory infiltrate in lamina propria, while in part, the lumen is covered by gastric mucosa with a moderate and, in some places, accentuated chronic inflammatory infiltrate in lamina propria with the presence of many eosinophils and multifocal mucinous metaplasia. There are small foci of cystic glandular dilation and an atrophic appearance of the mucosa. Parietal cells with a deeply eosinophilic cytoplasm are detected. In places, the inflammatory infiltrate spreads to the submucosa, where it has a much lesser intensity. In the part of the diverticular widening, there is thinning of the muscle layer of the wall of the diverticulum

Conclusion	The findings correlate with Meckel's diverticulum with gastric mucosa and signs of chronically active inflammation. The resection margin shows regular small intestinal structure except for the presence of a discrete inflammatory infiltrate in lamina propria

**Table 2:** Identified cases of Meckel's diverticulum treated with acid suppression.

Author and year	Presentation and diagnostic work-up	Medication	Outcome
1. Kirkpatrick A. R., 1978 [8]	A 27-year-old male with abdominal pain, gross bloody stool, and pallor, diagnosed using the Tc-99 m scan. The patient initially refused surgery due to financial reasons	Cimetidine 300 mg 4x/day per os.	Hemoglobin stabilized, and symptoms resolved for three months. Bleeding recurred upon stopping medication, and surgery was performed

2. Colins, J. C. Jr., 1980 [9]	A 26-year-old female with rectal bleeding and abdominal pain. Initial diagnosis was made with barium studies; it was confirmed with the Tc-99 m scan	Cimetidine initially i.v. and then per os.	There was no further bleeding. Elective surgery was performed 10 days later. Microscopic examination reiterated the diagnosis

3. Selker H. P., 1983 [10]	A 23-year-old male with postprandial infraumbilical pain, stool occult blood, and microcytic hypochromic anemia. Barium studies demonstrated a large ileal diverticulum; the Meckel scan was normal	Cimetidine 400 mg 4x/day per os.	Medication was stopped, and bleeding recurred. Cimetidine was restarted with good response, and surgery was performed five days later. Microscopic examination confirmed the diagnosis and demonstrated gastric-like mucosa

4. Xinias I. et al., 2012 [11]	An 8-year-old boy with bright red stools, eventually diagnosed with wireless capsule endoscopy	Ranitidine 6 mg/kg of body weight 2x/day per os.	Maintained symptom free for six months, after which surgery was performed. Microscopic examination confirmed the diagnosis

5. Dashan A., 2006 [12]	A 9-year-old boy with overt gastrointestinal bleeding. Diagnosis was made with isotope scanning	Pantoprazole 40 mg/day i.v.	Symptoms resolved, and surgery was performed 4 days after initial presentation

6. Ottaviano L. F. et al., 2016 [13]	A 72-year-old female with beta-thalassemia trait presented with weakness and anemia. Double-balloon enteroscopy found midileal diverticulum with adjacent ulcer	PPI	The patient refused surgery, and at 7 months' follow-up, hemoglobin remained stable with no new episodes of melena or overt GI bleeding

7. Ottaviano L. F. et al., 2016 [13]	An 80-year-old male with abdominal pain and anemia. CT showed distal small-intestinal wall thickening, and video capsule endoscopy demonstrated an ulcer. Double-balloon enteroscopy showed a nonbleeding jejunal angioectasia (treated with argon plasma coagulation) and ulcerated midileal mucosa surrounding a small diverticulum	PPI	Symptom resolution up to study date

8. Manning R. J., 1987 [14]	A 25-year-old male with copious gastrointestinal bleeding	Cimetidine 300 mg 4x/day and ranitidine 150 mg 3x/day per os.	Despite two H2 blockers and aggressive i.v. fluid and blood replacement, bleeding persisted, and emergent surgery was performed. Meckel's diverticulum was found intraoperatively and confirmed microscopically

## Data Availability

All data underlying the results are available as part of the article, and no additional source data are required.

## References

[1] Stallion A., Shuck J. M., Holzheimer R. G., Mannick J. A. (2001). Meckel’s diverticulum. *Surgical Treatment: Evidence-Based and Problem-Oriented*.

[2] Kuwajerwala N. K., Silva Y. J., Kanthimathinathan V. S., Villalba M. R., Mohammed A., Geibel J. (2021). *Meckel Diverticulum*.

[3] Hansen C. C., Søreide K. (2018). Systematic review of epidemiology, presentation, and management of meckel’s diverticulum in the 21st century. *Medicine*.

[4] Sagar J., Kumar V., Shah D. K. (2006). Meckel’s diverticulum: a systematic review. *Journal of the Royal Society of Medicine*.

[5] Kuru S., Kismet K. (2018). Meckel’s diverticulum: clinical features, diagnosis and management. *Revista Espanola de Enfermedades Digestivas: Organo Oficial de La Sociedad Espanola de Patologia Digestiva*.

[6] Kotha V. K., Khandelwal A., Saboo S. S. (2014). Radiologist’s perspective for the meckel’s diverticulum and its complications. *The British Journal of Radiology*.

[7] Pisegna J. R. (2002). Pharmacology of acid suppression in the hospital setting: focus on proton pump inhibition. *Critical Care Medicine*.

[8] Kirkpatrick R. A. (1978). Cimetidine and meckel’s diverticulum. *Annals of Internal Medicine*.

[9] Collins J. C. (1980). Hemorrhage from a meckel’s diverticulum: one case with heterotopic gastric mucosa treated with cimetidine. *Archives of Surgery*.

[10] Selker H. P. (1983). Cimetidine and cryptic “dyspepsia meckeli”. *JAMA: The Journal of the American Medical Association*.

[11] Xinias I., Mavroudi A., Fotoulaki M., Tsikopoulos G., Kalampakas A., Imvrios G. (2012 Sep). Wireless capsule endoscopy detects meckel’s diverticulum in a child with unexplained intestinal blood loss. *Case Reports in Gastroenterology*.

[12] Dahshan A. (2007). Bleeding meckel diverticulum responds to intravenous pantoprazole. *Southern Medical Journal*.

[13] Ottaviano L. F., Cui Y., Quintero E., Buscaglia J. M., Carlos J. (2016). Suspected meckel’s diverticulum as cause of gastrointestinal bleeding in elderly adults. *American Journal of Gastroenterology*.

[14] Manning R. J. (1987). Failure of H2 blocker therapy in a case of hemorrhage from a meckel’s diverticulum. *Journal of Clinical Gastroenterology*.

